# Enhancing Quantum Dot Photovoltaic Efficiency Through Defect Passivation and Triplet Energy Transfer with 9‐Anthracenecarboxylic Acid

**DOI:** 10.1002/smsc.202500306

**Published:** 2025-08-27

**Authors:** Eon Ji Lee, Gayoung Ham, Sunhee Yun, Hyung Ryul You, Taeyeong Yong, Gayoung Seo, Wonjong Lee, Hyeon Soo Ma, Jin Young Park, Hae Jeong Kim, Soo‐Kwan Kim, Younghoon Kim, Jongchul Lim, Minjun Kim, Hyojung Cha, Jongmin Choi

**Affiliations:** ^1^ Department of Energy Science and Engineering (ESE) Daegu Gyeongbuk Institute of Science and Technology (DGIST) Daegu 42988 Republic of Korea; ^2^ School of Energy Engineering Kyungpook National University (KNU) Daegu 41566 Republic of Korea; ^3^ Department of Chemical Engineering Pohang University of Science and Technology (POSTECH) Pohang 37673 Republic of Korea; ^4^ Graduate school of Energy Science and Technology (GEST) Chungnam National University (CNU) Daejeon 34134 Republic of Korea; ^5^ Department of Chemistry Kookmin University (KMU) Seoul 02707 Republic of Korea; ^6^ Department of Chemistry Kyonggi University (KGU) Suwon 16227 Republic of Korea

**Keywords:** defect passivation, PbS colloidal quantum dots, photovoltaics, triplet energy transfer, zinc oxide

## Abstract

A dual‐functional electron transport layer (ETL) is reported for PbS colloidal quantum dot (CQD) photovoltaics by incorporating 9‐anthracenecarboxylic acid (ACA) into a zinc oxide (ZnO) matrix. Despite its favorable electron transport characteristics and appropriate band alignment, intrinsic defects in ZnO, such as oxygen vacancies, remain a limiting factor in device performance. The carboxylate functional group of ACA effectively passivates these defects, thereby reducing trap‐assisted recombination. Moreover, ACA, an acene‐based π‐conjugated molecule, efficiently generates triplet excitons. These triplets undergo triplet energy transfer to the PbS CQD layer, enhancing photocurrent generation. Owing to these synergistic effects, CQD photovoltaics (PVs) incorporating ACA‐treated ZnO ETLs exhibit enhanced open‐circuit voltage and short‐circuit current density, resulting in a higher power conversion efficiency of 11.55% compared to 10.48% for control devices. This strategy highlights the combined advantages of electronic defect passivation and triplet exciton harvesting in PbS CQD PVs.

## Graphical abstract

An acene‐based donor incorporated into the electron transport layer enables triplet energy transfer to PbS colloidal quantum dots and improves defect passivation, resulting in enhanced photovoltaic device performance.

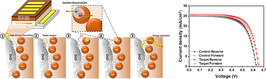

## 1 Introduction

PbS colloidal quantum dot (CQDs) have been extensively studied as photovoltaic (PV) absorbers due to their broad absorption spectrum (ranging from ultraviolet to short‐wavelength infrared), high absorption coefficient, large exciton Bohr radius (18 nm), low exciton binding energy (≈20 meV), and excellent solution processability.^[^ ^1^ ^]^ Since Sargent et al. first introduced PbS CQDs into PVs in 2005, a certified power conversion efficiency of PbS CQD PVs has reached 13.40% over the past 20 years, driven by advances in CQD surface chemistry and device architecture.^[^ ^2^, ^3^, ^4^, ^5^, ^6^, ^7^ ^]^ More recently, owing to their superior infrared absorption and compatibility with low‐temperature processing, PbS CQD PVs have shown great promise as next‐generation PV components for tandem device applications.^[^ ^8^, ^9^, ^10^ ^]^

In PbS CQD PVs, zinc oxide (ZnO) is widely employed as the electron transport layer (ETL) due to its excellent electron affinity, optical transparency, and favorable conduction band alignment with PbS CQDs.^[^ ^11^, ^12^, ^13^ ^]^ However, ZnO contains intrinsic defects, such as oxygen vacancies, which act as trap sites (recombination centers) and ultimately limit the open‐circuit voltage (*V*_OC_) and short‐circuit current density (*J*_SC_) of the device.^[^ ^13^, ^14^, ^15^, ^16^ ^]^ These defect sites not only promote nonradiative recombination but also disrupt the energetic landscape at the ZnO/PbS CQD interface, impeding efficient charge transfer.

To mitigate such defects, recent studies have investigated surface passivation strategies using organic molecules containing functional groups capable of binding to undercoordinated zinc or oxygen atoms in ZnO.^[^ ^17^, ^18^ ^]^ Among these, benzoic acid, which includes a carboxyl group, has been shown to form strong bonds with Zn^2+^ ions, effectively reducing nonradiative trap states at the interface, as demonstrated with other carboxyl‐based materials.^[^ ^19^ ^]^ Inspired by these findings, we incorporate ACA, which also possesses the carboxyl group, into the ZnO sol–gel to chemically interact with undercoordinated sites, thereby achieving surface passivation of the ZnO film and potentially introducing additional synergistic effects in CQD PVs.

Beyond defect passivation, ACA—an acene‐based conjugated organic molecule—also offers the potential for triplet energy transfer (TET) from the ETL to the CQD layer. The ACA has a planar π‐conjugated structure, and its triplet energy (≈1.83 eV) exceeds the bandgap of PbS quantum dots (≈1.36 eV), enabling energetically favorable TET.^[^ ^20^, ^21^, ^22^ ^]^ Triplet excitons exhibit significantly longer diffusion lengths and lifetimes than singlet excitons,^[^ ^23^, ^24^ ^]^ which may contribute to enhanced PV performance. However, due to their spin‐forbidden nature, triplet excitons have rarely been utilized in bulk semiconductor systems, as their harvesting typically requires complex material designs.^[^ ^25^, ^26^ ^]^ In contrast, CQDs possess ill‐defined quantum numbers, and their excited states are separated by much smaller energy gaps (Δ*E*_ST_, 1–15 meV), compared to typical organic semiconductors (≈100 meV), thereby enabling TET with minimal energy loss.^[^ ^20^, ^27^, ^28^ ^]^ Previous studies demonstrated that PbS CQDs can serve as efficient triplet acceptors.^[^ ^29^ ^]^ For instance, in 2012, Greenham et al. reported a PV device with a power conversion efficiency (PCE) of 0.85% that harvested triplet excitons using bilayer films of pentacene and PbS CQDs.^[^ ^30^ ^]^ In a follow‐up study in 2015, the same group achieved a PCE of 4.8% using TIPS‐pentacene and narrow‐bandgap PbS CQDs, experimentally verifying that PbS CQDs can dissociate triplet excitons into free charges.^[^ ^31^ ^]^

Based on these findings, in this study, we embed ACA into the ZnO ETL, utilizing it as a dual‐functional material that simultaneously passivates ZnO defects and serves as a triplet donor to the PbS CQD active layer. This integrated design leverages both electronic and photonic synergies to address the limitations of conventional ZnO/PbS CQD interfaces. We systematically investigated the optoelectronic and PV performance of PbS CQD PVs incorporating the ZnO–ACA ETL. The introduction of the ACA led to a reduction in oxygen vacancies in ZnO via passivation through its carboxyl group. In addition, we demonstrated that ACA can effectively generate triplet excitons and transfer them to CQD solids, thereby contributing to improved photocurrent density. These properties resulted in enhanced device performance, with an increase in *V*_OC_ (from 0.629 to 0.654 V) and *J*_SC_ (from 25.02 to 25.83 mA cm^−2^), ultimately yielding a higher PCE of 11.55% compared to the control device (10.48%).

## 2 Results and Discussion

To investigate the effect of ACA, we prepared ACA‐treated ZnO sol–gel solutions by adding ACA at various concentrations (0.1 to 1.0 mg mL^−1^) after synthesizing the ZnO sol–gel. The samples prepared by mixing ZnO sol–gel solutions with ACA at concentrations of 0.1, 0.3, 0.5, 0.75, and 1.0 mg mL^−1^ are referred to as ACA‐0.1, ACA‐0.3, ACA‐0.5, ACA‐0.75, and ACA‐1.0, respectively. The solutions were drop‐cast onto indium tin oxide (ITO) substrates and spin‐coated at 4,000 rpm. Immediately after coating, the films were annealed at 200 °C for 30 min to induce ZnO crystallization.

**Figure 1:**
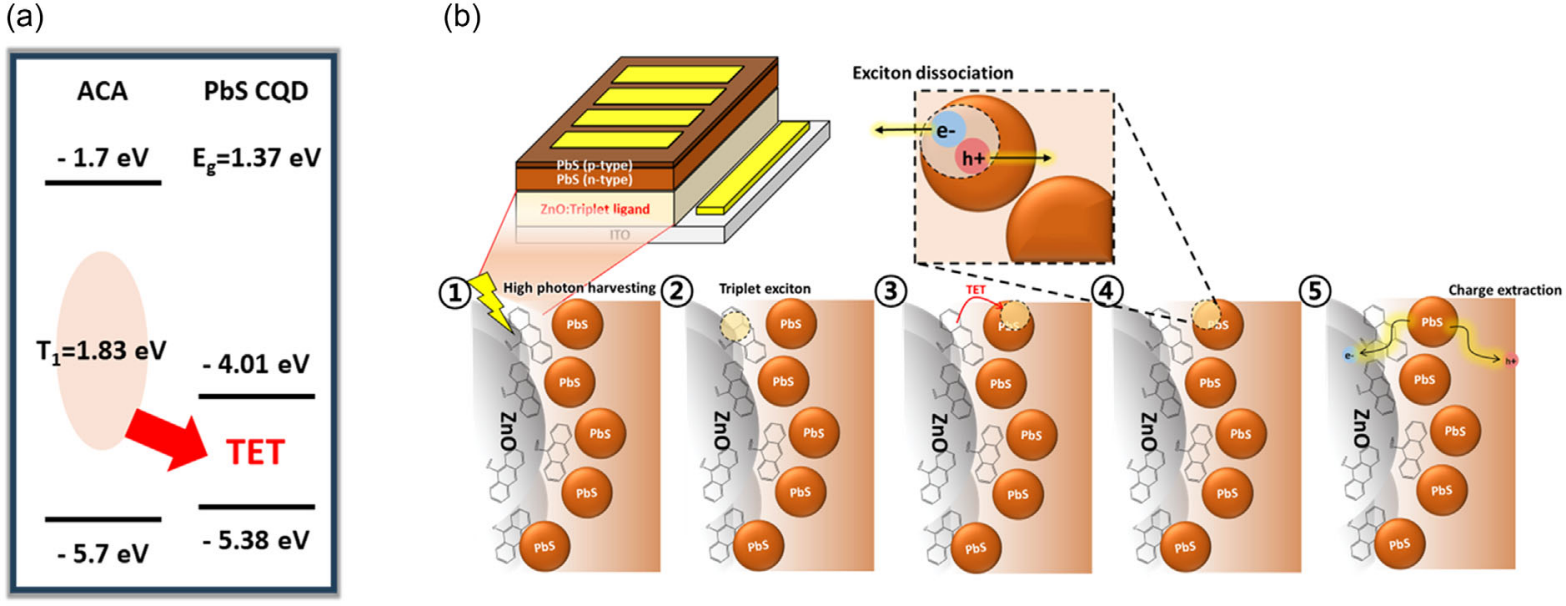
a) Schematic energy level diagram of ACA and PbS CQDs, showing the proposed TET process (red arrow). b) Schematic illustration of the TET mechanism in PbS CQD PVs.

**Figure** **1**a illustrates the energy band alignment between ACA and PbS CQDs.^[^ ^32^, ^33^ ^]^ In this study, PbS CQDs with a bandgap of ≈1.37 eV were used (Figure S1, Supporting Information). For an efficient TET mechanism, appropriate energy level alignment between materials is essential. In particular, the triplet donor must exhibit a cascaded energy level configuration with the PbS CQDs, and the ACA/PbS CQD system satisfies the condition for charge‐ transfer‐mediated TET.^[^ ^32^ ^]^ ACA possesses the triplet energy of 1.83 eV, while the bandgap of PbS CQDs is 1.37 eV, ensuring energetically favorable TET. To evaluate the effect of ACA on the energy band alignment of the ZnO ETL, we conducted ultraviolet photoelectron spectroscopy (UPS) measurements on ZnO films blended with various concentrations of ACA, as shown in Figure S2 and Table S1 (Supporting Information). The analysis revealed a slight upward shift in the energy levels, including minor changes in the Fermi level position. However, these shifts were not significant enough to substantially hinder carrier extraction. Figure 1b illustrates the proposed mechanism of TET operation in the ACA‐treated PbS CQD PV system. Upon ultraviolet light absorption by the ACA‐blended ZnO film, ACA generates triplet excitons. These triplet excitons are then transferred to the PbS CQD solids at the ZnO/PbS CQD interface. Subsequently, the triplet excitons are dissociated into free charges within the PbS CQD layer and contribute to photocurrent generation.

**Figure 2:**
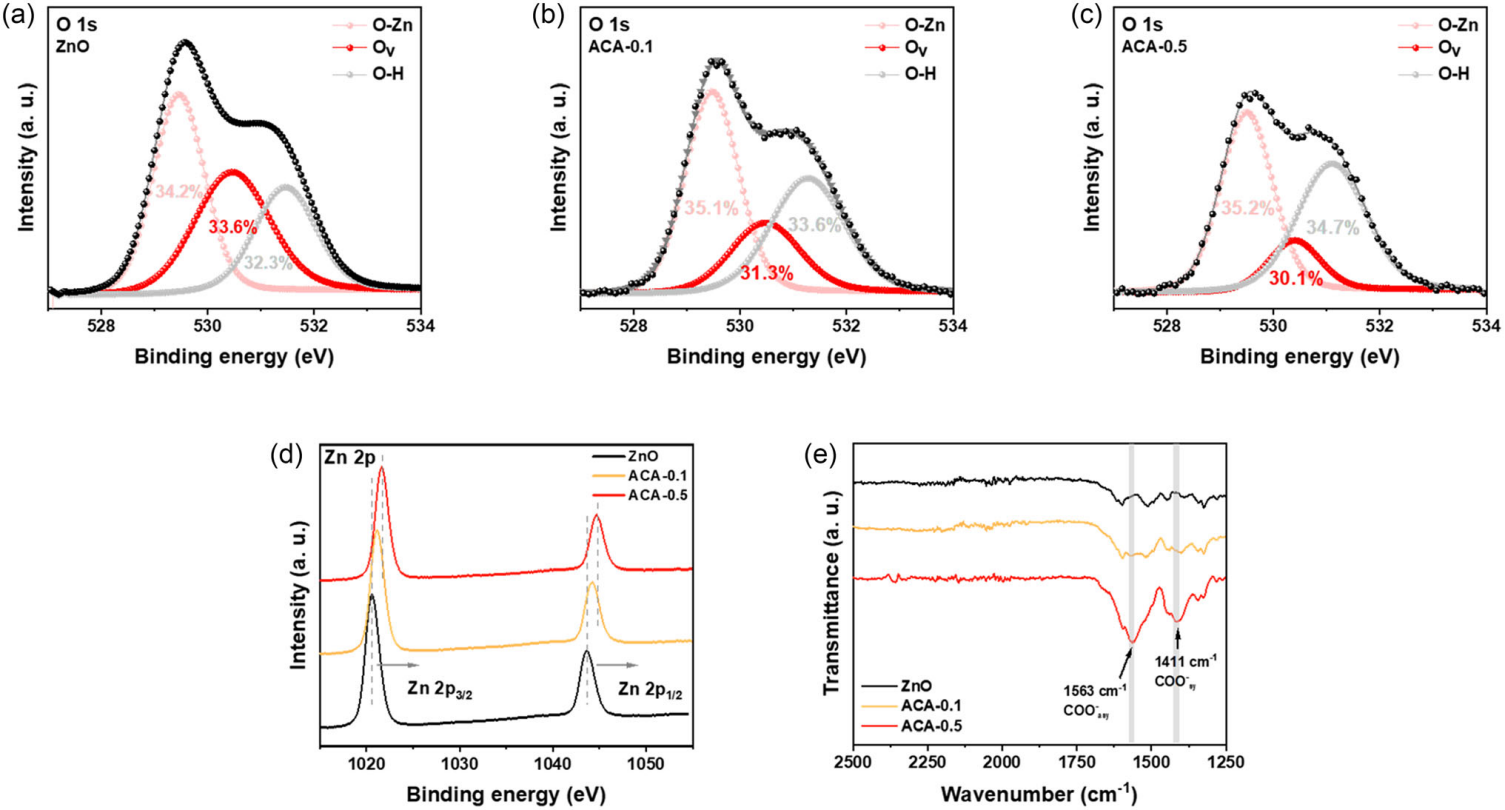
XPS spectra of a) pristine, b) ACA‐0.1, and c) ACA‐0.5 ZnO films. d) Zn 2p XPS spectra and e) FTIR spectra of ZnO, ACA‐0.1, and ACA‐0.5 films. The gray lines in the FTIR graph indicate the symmetric and asymmetric vibrational modes of the COO^−^ group, respectively.

To evaluate the passivation effect of ACA, we conducted a systematic analysis of control and ACA‐treated devices using X‐ray photoelectron spectroscopy (XPS). **Figure** **2**a presents the O 1*s* spectrum of the pristine ZnO film. Typically, the O 1*s* peak of ZnO can be deconvoluted into three components: the low binding energy peak at 530.5 eV corresponds to lattice oxygen in O–Zn bonding, while the higher binding energy peaks at 531.5 and 532.5 eV are attributed to oxygen vacancies and hydroxyl (–OH) groups, respectively.^[^ ^34^, ^35^ ^]^ After ACA treatment, the relative intensity of the higher binding energy components decreased (Figure 2b,c), indicating a reduction in surface oxygen‐deficient defects. Specifically, analysis of the relative proportions of O—Zn bonds, oxygen vacancies, and hydroxyl groups revealed that the pristine ZnO film exhibited an oxygen vacancy ratio of 33.6%. This ratio gradually decreased to 31.3% for the ACA‐0.1 film and 30.1% for the ACA‐0.5 film, indicating a progressive reduction in oxygen vacancies with ACA treatment. This suggests that the carboxylate groups in ACA effectively passivate ZnO surface defects, which are known to serve as major charge recombination centers at the ZnO/PbS CQD interface. The Zn 2p XPS spectra shown in Figure 2d reveal a binding energy shift from 1020.58 to 1021.59 eV following ACA treatment, indicating the formation of chemical bonds between ZnO and ACA.^[^ ^36^ ^]^ Furthermore, Fourier‐transform infrared (FTIR) analysis (Figure 2e) shows that the intensity of peaks corresponding to carboxylate (–COO^−^) vibrations—1563 cm^−1^ for the asymmetric mode and 1411 cm^−1^ for the symmetric mode—increased with higher ACA concentrations, confirming the successful binding of ACA to the ZnO surface.^[^ ^37^ ^]^

To further investigate the physical changes induced by the coordination between the carboxylate group of ACA and Zn^2+^ ions, we measured the top‐view morphology of films prepared under ZnO, ACA‐0.1, and ACA‐0.5 conditions using scanning electron microscopy (SEM). Figure S3 and S4 (Supporting Information) were taken with 100 and 10 μm scale bars, respectively, to provide different magnifications. As shown in Figure S4a, Supporting Information, the pristine ZnO film contained a significant number of pinholes on the surface. In contrast, the ACA‐0.1 film (Figure S4b, Supporting Information) displayed a notable reduction in pinhole density, although some non‐uniform regions still remained. Remarkably, the ACA‐0.5 film (Figure S4c, Supporting Information) showed the most uniform surface morphology, with diminished surface irregularities and pinhole formation. This observation aligns with previous reports showing that various dicarboxylic acids can form chelate complexes with Zn^2+^ ions in ZnO, thereby influencing particle growth depending on the acid species.^[^ ^38^ ^]^ We therefore infer that ACA facilitates more uniform ZnO growth, resulting in a smoother and more compact film surface.

Additionally, due to the high solubility of ACA in polar solvents such as butylamine (the solvent used for the PbS‐halide CQD ink), we investigated whether residual ACA in the ACA‐blended ZnO layer chemically interacts with the overlying PbS CQDs by conducting absorption measurements (Figure S5, Supporting Information). To prepare the samples, ZnO or ACA‐blended ZnO was first spin‐coated onto the substrate, followed by spin‐coating a PbS‐halide CQD ink dispersed in butylamine (BTA) on top. As shown in Figure S5, Supporting Information, the absorption spectra of the PbS CQD films deposited on pristine ZnO and ACA‐0.5 were nearly identical to that of a standalone PbS CQD film. This result suggests that no additional chemical interaction occurs between the residual ACA in the ETL and the surface of the PbS CQDs under the processing conditions used.

To verify the effect of ACA on PbS CQD PVs and determine the optimal ACA concentration for device performance, space charge limited current (SCLC) measurements were performed using electron‐only devices with the structure ITO/ETL/PbS‐halide/Au.^[^ ^39^ ^]^ The electron trap‐state densities (*N*_t_) were determined from dark *I–V* curves plotted on a logarithmic scale. The applied voltage at the kink point between the ohmic region (*n* = 1) and the trap‐filled region (*n* > 3), referred to as the trap‐filled limit voltage (*V*_TFL_), was used to calculate *N*_t_ based on film thickness (Figure S6, Supporting Information), following a previously reported equation.^[^ ^40^ ^]^ The control devices exhibited a trap density of 7.86 × 10^1^ ^5^ cm^−3^, while the ACA‐0.1 and ACA‐0.5 devices showed reduced values of 6.55 × 10^1^ ^5^ and 5.24 × 10^1^ ^5^ cm^−3^, respectively (Figure S7 and Table S2, Supporting Information). These results confirm that the incorporation of ACA effectively suppresses ZnO‐related trap states.

However, when the ACA concentration exceeded 0.5 mg mL^−1^, decreases in both *V*_OC_, and *J*_SC_ were observed. This performance degradation was likely attributed to unfavorable energy level alignment and the presence of excess ACA that did not bind to the ZnO surface, acting as an impurity and potentially reducing the electrical conductivity of the ETL (Figure S8, Supporting Information). Therefore, the optimal ACA concentration was determined to be 0.5 mg mL^−1^, and all subsequent devices were fabricated using ZnO ETLs incorporating ACA at this concentration.

Additionally, to evaluate the conductivity of the control (ZnO) and target (ACA‐0.5) films, we fabricated ETL‐only devices and conducted conductivity measurements (Figure S9, Supporting Information). For this, ZnO and ACA‐blended ZnO sol–gel solutions were respectively spin‐coated onto ITO substrates, followed by thermal annealing at 200 °C for 30 min. Subsequently, an 80 nm‐thick Au layer was deposited to serve as the anode. The measurement results showed that the ACA‐blended ZnO exhibited slightly higher conductivity compared to the pristine ZnO control, which can be attributed to ACA‐induced defect passivation that reduces charge recombination.

**Figure 3:**
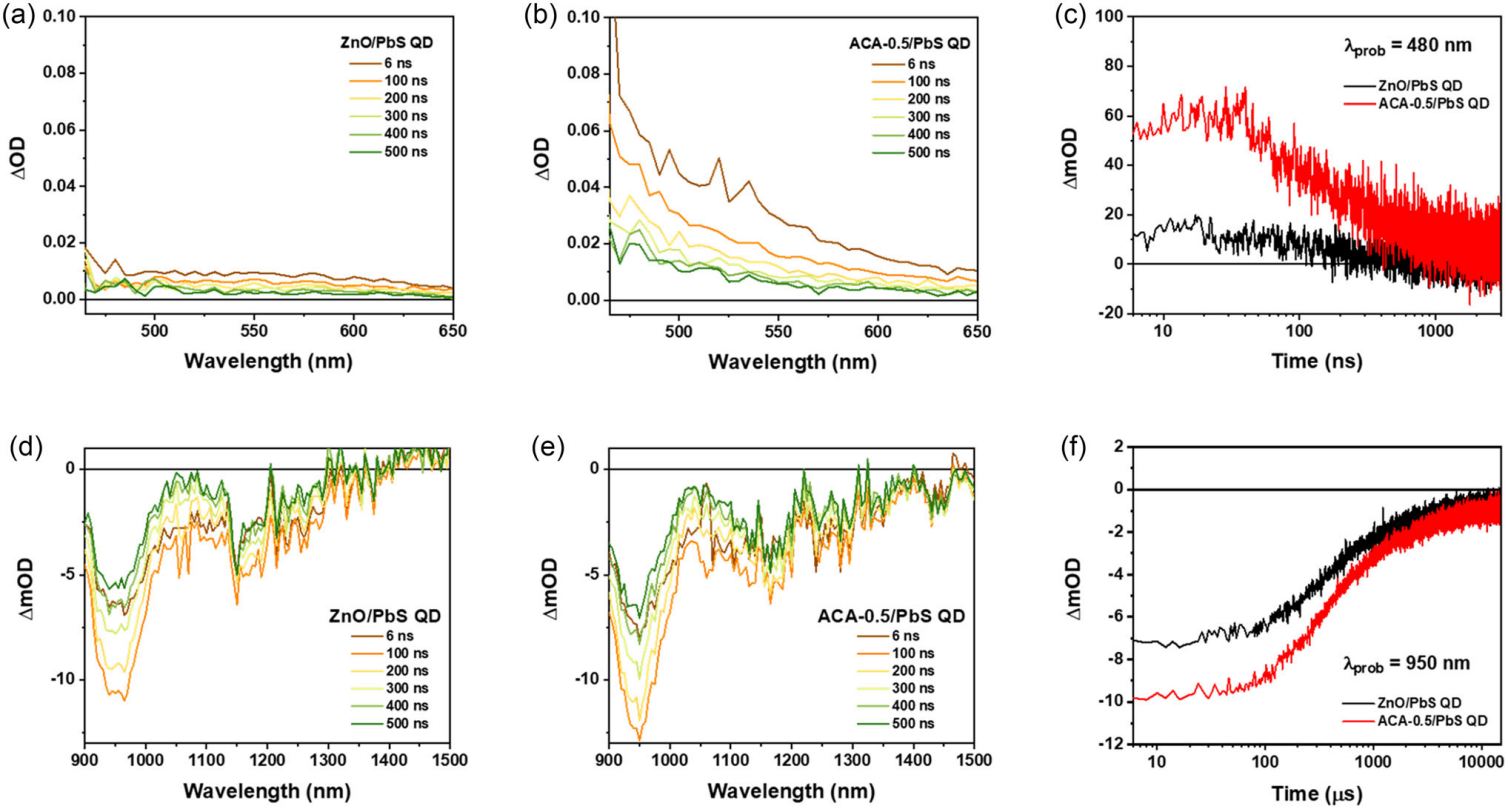
Transient absorption spectra of a) ZnO/PbS CQD bilayer and b) ACA‐0.5/PbS CQD bilayer films in the visible region. Time delays range from 6 ns (brown) to 500 ns (green). c) Transient absorption kinetics of the ZnO/PbS CQD bilayer probed at 480 nm. Transient absorption spectra of d) ETL/PbS CQD bilayer and e) ACA‐0.5/PbS CQD bilayer films in the NIR. f) Transient absorption kinetics of the ETL/PbS CQD bilayer probed at 950 nm.

We also investigated the optical effect of ACA treatment on ZnO by measuring its absorption and transmittance spectra, as shown in Figure S10 and S11 (Supporting Information). The absorption profile of the ZnO film was consistent with values reported in previous literature, and the ACA‐treated ZnO film exhibited generally increased absorbance in its bandgap region compared to the pristine ZnO film.^[^ ^4^ ^]^ We further conducted transmittance measurements to assess whether incident light could penetrate the PbS CQD layer. Although a slight decrease in transmittance was observed due to light absorption by ACA molecules, the overall transmittance of the ACA‐0.5 film remained comparable to that of the pristine ZnO film and did not significantly hinder light penetration. Subsequently, we investigated the triplet‐state dynamics induced by the incorporation of ACA into the ZnO using transient absorption spectroscopy (TAS) across the ultraviolet–visible (UV) and near‐infrared regions (NIR, **Figure** **3**a,b and S12, Supporting Information). To ensure sufficient photoexcitation of the ACA, a xenon lamp was used as the probe source, and pump pulses were applied to the films at wavelengths of 355 and 532 nm, respectively. In the visible region (465–650 nm), a significantly enhanced photoinduced absorption (PIA) signal was observed under the ACA‐0.5 condition (Figure 3b) compared to the pristine ZnO film (Figure 3a). This enhancement is attributed to the formation of ACA triplet excitons via the TET, as supported by the slower decay dynamics. Although the T_1_ → T_n_ transition of ACA is known to occur around 430 nm wavelength, spectral overlap between ground‐state bleach (GSB) and PIA at this wavelength (Figure S12c, Supporting Information) hindered clear analysis.^[^ ^29^ ^]^ Therefore, we examined the charge carrier dynamics at 480 nm to isolate the PIA signal without interference from GSB (Figure 3c). Upon ACA treatment, the charge carrier lifetime increased from 274 to 374 ns. This result indicates that charge carriers at the ZnO/PbS CQD interface are effectively transferred to ACA‐induced triplet states, thereby suppressing interfacial charge recombination (Figure S13 and Table S3, Supporting Information). In contrast, at the interface between pristine ZnO and PbS CQDs, rapid recombination of photoexcited carriers occurs due to the absence of such intermediate energy states. These findings suggest that the ACA plays a critical role in enhancing charge extraction at the ZnO/PbS CQD interface by introducing intermediate states that delay recombination, thereby prolonging charge carrier lifetimes and potentially improving device performance. In the near‐infrared region, the GSB of PbS CQDs centered around 950 nm was observed (Figure 3d,e). The gradual decay from 6 to 500 ns, along with sustained charge generation over this period, indicates the excellent charge generation capability of the PbS CQDs. The corresponding GSB kinetics were analyzed and are presented in Figure 3f. Upon ACA treatment, significantly longer recombination lifetimes were observed, increasing from 825.5 to 995.3 ns (Figure S14 and Table S4, Supporting Information). This increase in GSB is attributed to the efficient transfer of triplet excitons generated in the ACA moieties to the PbS CQDs (Figure 3e). In contrast, in the absence of ACA, the ZnO/PbS CQD interface exhibited a faster recombination process, suggesting that the excited carriers recombined directly without intermediate state trapping. These findings support a delayed recombination mechanism that aligns with the slow triplet‐state formation kinetics of ACA.

To evaluate the effect of ACA molecules on the charge recombination and charge extraction (separation) dynamics at the ETL/PbS quantum dot interface, we conducted transient photovoltage (TPV) and transient photocurrent (TPC) measurements with the full device structure. Figure S15a,b (Supporting Information) show that the target device exhibited an extended carrier lifetime of 3.09 μs, whereas the control device showed a shorter carrier lifetime of 2.43 μs. The extended carrier lifetime observed in the ACA‐0.5 device suggests a significant reduction in charge recombination, due to the carboxylate groups of ACA effectively passivating oxygen vacancies within the ZnO layer. This results in fewer electronic trap states at the ZnO, thereby improving carrier dynamics compared to the control devices. Furthermore, we conducted the TPC measurements as shown in Figure S15c,d (Supporting Information). The target device exhibited a faster carrier extraction time of 1.30 μs, compared to 1.44 μs for the control device with pristine ZnO. This enhancement can be attributed to effective defect passivation within the ZnO layer by ACA, which facilitates smoother charge extraction compared to the control condition. In addition, it was confirmed that triplet states generated from the ACA successfully migrated into the PbS‐halide active layer, where efficient separation into electrons and holes occurred, followed by effective extraction into the ETL and hole transport layer (HTL), respectively. This indicates that the excitons generated from the ACA migrated smoothly through the active layer and that charge transport and extraction toward the electrodes proceeded favorably.

**Figure 4:**
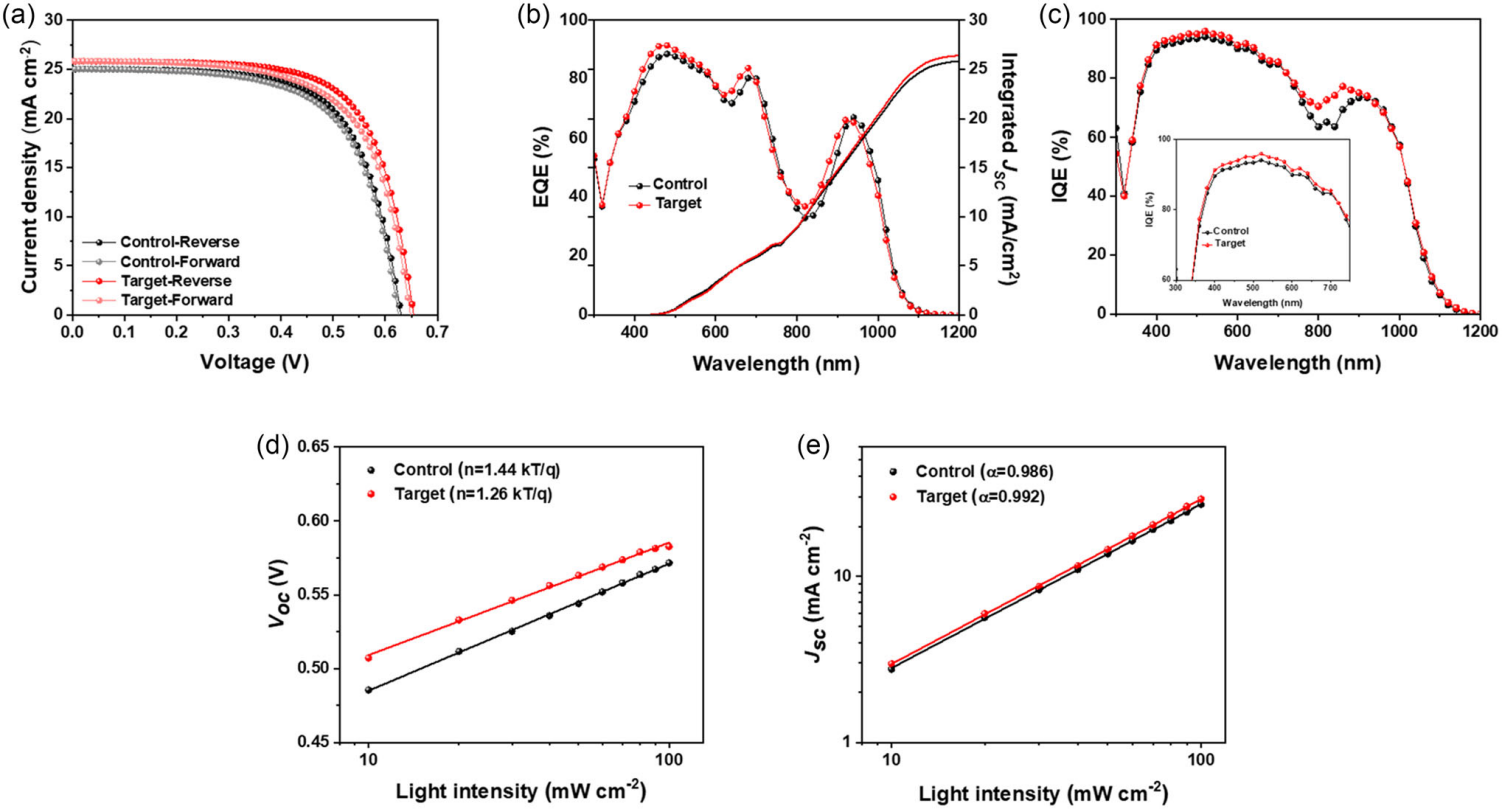
Device performance of PbS CQD PVs. a) *J–V* characteristics under AM 1.5 G illumination (100 mW cm^−2^), b) EQE, and c) IQE spectra. The inset in (c) shows a magnified view of the IQE curve. d) Light intensity‐dependent *V*_OC__,_ and e) light intensity‐dependent *J*_SC_ characteristics.

The advantages of ACA‐treated ZnO over conventional ZnO are evident in the performance comparison of CQD PV devices. The device architecture consisted of ITO/ZnO (with or without ACA)/PbS–PbX_2_ (X = I, Br)/PbS–1,2‐Ethanedithiol (EDT)/Au. **Figure** **4**a presents the *J*–*V* curves of the champion devices based on pristine ZnO and ACA‐0.5. Detailed PV parameters are summarized in **Table** **1**. The target device achieved a PCE of 11.55%, whereas the device with untreated ZnO exhibited a PCE of 10.48%. The enhanced PCE of the ACA‐treated device is attributed to improvements in *V*_OC_ from 0.629 to 0.654 V, *J*_SC_ from 25.02 to 25.83 mA cm^−^^2^, and fill factor (FF) from 66.53% to 68.42%. These enhancements can be attributed to improved ZnO defect passivation and the contribution of the TET effect. A similar trend was consistently observed in the histogram shown in Figure S16, Supporting Information. Figure 4b presents the external quantum efficiency (EQE) spectra of devices based on ZnO and ACA‐0.5. The *J*_SC_ values calculated from the EQE measurements were in good agreement with those obtained from the *J*–*V* curves (Table 1). To assess how efficiently absorbed photons contributed to the current generation, internal quantum efficiency (IQE) measurements were also conducted (Figure 4c). The results revealed an increase in IQE across the spectral ranges of 350–700 nm and 740–920 nm. This enhancement can be attributed to improved electron collection and transport in the ACA‐treated film, which exhibits reduced trap densities. Additionally, long‐term stability tests were performed for devices based on ZnO and ACA‐0.5 (Figure S17, Supporting Information), and the ACA‐treated devices demonstrated comparable stability to the control device under ambient conditions over a period of 33 days. In addition, to investigate the stability of the control and target devices under high‐temperature and high‐humidity conditions, full devices were exposed to an environment of 85 °C and 85% relative humidity (RH), as shown in Figure S18, Supporting Information. As a result, in the early stage, the oxidation of the HTL (PbS capped with EDT) led to a reduction in quantum dot size and a widening of the bandgap, which enhanced the electron blocking effect and resulted in a slight increase in PCE.^[^ ^41^ ^]^ However, as oxidation progressed over time in both the control and target devices, an insulating layer gradually formed, leading to a decrease in PCE. Ultimately, both devices exhibited a similar level of PCE degradation.

**Table 1:** PV parameters of the best‐performing PbS CQD PVs with and without ACA treatment. The calculated *J*_SC_ values are determined from the EQE results (Figure 4b).

Type	*V*_OC_ [V]	*J*_SC_ [mA cm^−2^]	FF [%]	PCE [%]	Calculated *J*_SC_ from EQE [mA cm^−2^]
Control (Reverse)	0.629	25.02	66.53	10.48	25.78
Control (Forward)	0.625	25.01	64.93	10.15	
Target (Reverse)	0.654	25.83	68.42	11.55	26.46
Target (Forward)	0.648	25.83	65.29	10.92	

To gain deeper insights into the effect of the ACA on device performance, light intensity‐dependent characteristics were investigated. The *V*_OC_ was plotted as a function of light intensity using a previously reported equation (Figure 4d).^[^ ^33^ ^]^ The slope (*n*) of the plot for the ACA‐treated devices was significantly lower (1.26 kTq^−1^) than that of the control devices (1.44 kTq^−1^), indicating that ACA reduces trap‐assisted recombination and contributes to improved *V*_OC_. The log–log plot of *J*_SC_ versus light intensity is shown in Figure 4e. The α values for the ZnO and ACA‐0.5 devices were 0.986 and 0.992, respectively—both close to unity. This result suggests that bimolecular recombination is negligible in both devices.

**Figure 5:**
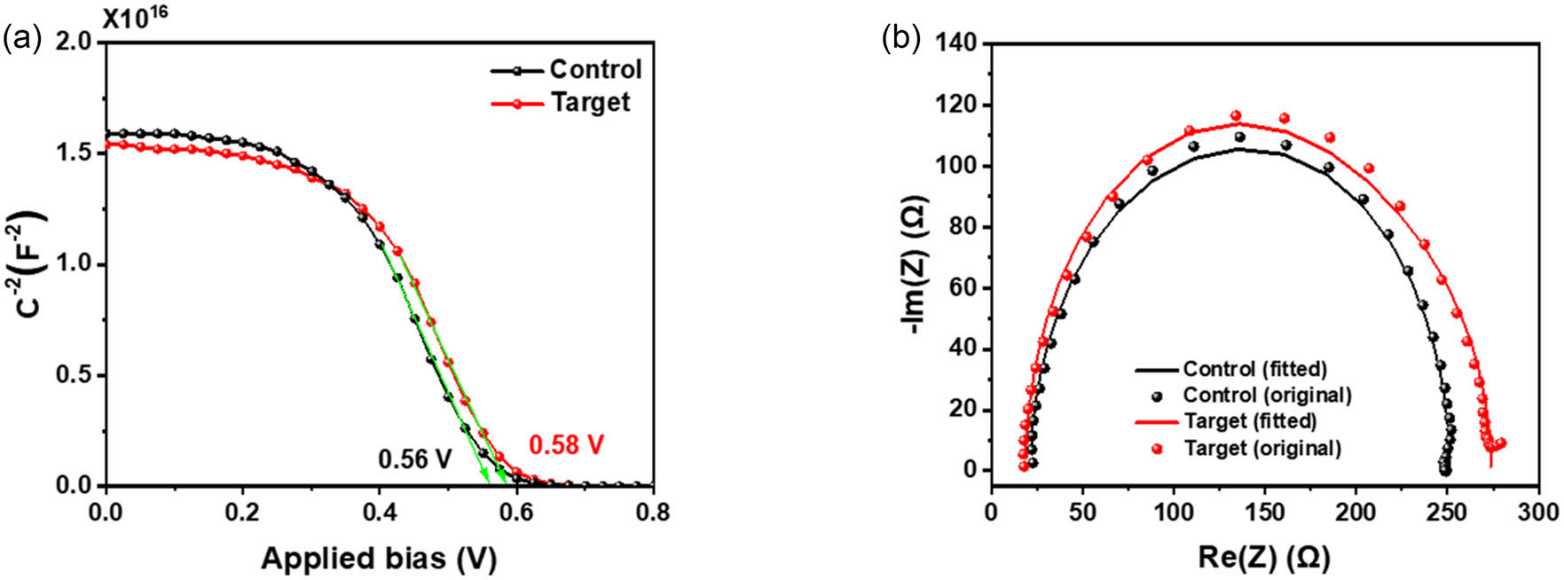
a) Mott–Schottky analysis of the control and target devices with a frequency at 200 kHz. b) Nyquist plots of PbS CQD PVs of control and target conditions.

We also investigated the Mott–Schottky characteristics of both the control and target devices (**Figure** **5**a). The measurements were conducted on full devices with the structure: ITO/ETL/PbS‐PbX_2_ (X = I, Br)/PbS‐EDT/Au. As shown in the Mott–Schottky plot, the built‐in potential (*V*_bi_) of the ACA‐blended target device was 0.58 V, and the *V*_bi_ value of the control device was 0.56 V, consistent with the observed improvement in *V*_OC_. A stronger built‐in electric field promotes more efficient charge separation and transport, resulting in suppressed recombination and enhanced *V*_OC_. Additionally, electrochemical impedance spectroscopy (EIS) was performed to further probe the charge dynamics. Full devices were prepared under both control and target conditions, and the measurements were conducted under 0.8 sun illumination (Figure 5b). As a result, the series resistance in the target device was measured to be 18.61 Ω, which is lower than that of the control device (21.40 Ω). Additionally, the charge transfer resistance decreased from 82.59 Ω (control) to 57.70 Ω (target). And the recombination resistance increased from 147.11 Ω (control) to 197.50 Ω for the target condition (Table S4, Supporting Information). These results provide additional evidence that the suppression of charge recombination contributes to the observed improvement in *V*_OC_.

## 3 Conclusion

In conclusion, we demonstrated that incorporating ACA into the ZnO ETL effectively enhances the performance of PbS CQD PVs by improving interfacial properties and enabling favorable photophysical interactions. The ACA‐treated ZnO exhibited a reduced trap‐state density, which led to enhanced charge extraction and suppressed interfacial recombination. Moreover, TAS and IQE measurements further supported the occurrence of TET from ACA to PbS CQDs, contributing to increased photocurrent generation. The best‐performing ACA‐based device achieved the PCE of 11.55%, surpassing that of the control device (10.48%). These findings suggest that triplet‐active surface engineering using conjugated organic molecules such as ACA represents a promising strategy for interfacial optimization and performance enhancement in next‐generation CQD PV devices.

## 4 Experimental Section

4.1

4.1.1

##### Synthesis and Purification of PbS CQD

PbS CQDs with the first excitonic peak at 915 nm (1.37 eV) were synthesized as reported in the method, with a modified version.^[^ ^42^ ^]^ Lead (II) acetate trihydrate (0.7647 g), oleic acid (1.5 mL), and octadecene (15 mL) were mixed in a three‐neck flask and heated to 100 °C under vacuum overnight, and then filled with N_2_. After the flask was filled with Ar gas, the temperature was raised to 95 °C. A stock solution, 0.21 mL of hexamethyldisilathiane dissolved in 10 mL of octadecene, was then injected rapidly into the flask for PbS CQD synthesis. The solution was cooled down to room temperature slowly. After that, the CQD solution was precipitated and then redispersed in toluene. The CQD was further purified twice by adding a mixture of acetone and toluene. Finally, the CQD was dissolved in octane (50 mg mL^−1^).

##### Preparation of ZnO by Sol–Gel Method and ACA‐Blended ZnO Sol–Gel

ZnO sol–gel synthesis was based on a modification of a previously reported method.^[^ ^11^ ^]^ 1 g of zinc acetate dihydrate and 0.284 mL monoethanolamine were dissolved in 10 mL of 2‐methoxyethanol solution. The mixture was stirred for 12 h at 60 °C. The solution was filtered through a 0.2 μm PTFE filter and then deposited onto the ITO substrate by spin‐coating at 4,000 rpm for 30 s. The as‐deposited film was then annealed at 200 °C for 30 min. For the ACA‐treated ZnO preparation, ACA was added to the sol–gel solution with different concentrations.

##### Ligand Exchange and Fabrication of PbS CQD PVs

The solution**–**phase ligand exchange (SPLE) process was carried out under ambient conditions. A ligand precursor solution was prepared in a glovebox by dissolving lead halides (0.1 M of lead iodide and 0.02 M of lead bromide), ammonium acetate (0.06 M), and potassium iodide (0.02 M) in N,N‐Dimethylformamide (DMF). Subsequently, 10 mL of crude PbS CQD solution (a mixture of 3 mL crude solution and 7 mL octane) was added to 10 mL of the ligand precursor solution in a 50 mL conical tube. The mixture was vigorously stirred for 2 min until the CQDs were fully transferred from the octane to the DMF phase. Then the upper octane phase was discarded, and the CQD‐containing DMF phase was washed four times with fresh octane to remove residual ligands and impurities. Following the ligand exchange, the PbS CQDs were precipitated by the addition of toluene and collected via centrifugation. The resulting CQD pellet was dried under vacuum for 20 min, and subsequently redispersed in butylamine at a concentration of 250 mg mL^−1^ for active layer deposition.

##### Characterization and Measurement

The UV–vis–NIR absorption spectra were measured using a JASCO V‐770 spectrophotometer. XPS spectra of ZnO films were measured on the ITO substrate. XPS was performed in a PHI5500 Multi‐Technique system using monochromatic Al‐Kα radiation (hν = 1486.7 eV). *J*−*V* characteristics were measured using a Keithley 2400 and an xenon lamp (450 W) with an AM 1.5 G filter. The devices were measured in reverse scan (0.7 to −0.1 V) and forward scan (–0.1 to 0.7 V) with 0.02 V s^−1^ of scan rate under AM 1.5 G illumination of 100 mW cm^2^ (Newport Oriel Sol 3A solar simulator), which was calibrated with a Quartz filter certified by National Renewable Energy Laboratory (NREL). The active area of the device was 0.0512 cm^2^, which was determined by the mask placed in front of the device. EQE and IQE spectra were measured using a Newport Oriel QuantX‐300 and Oriel Cornerstone 130 monochromator. EQE measurements were taken in the 300−1200 nm wavelength range with a 20 Hz chopper and 10 nm wavelength spacing. Mott–Schottky curves were measured using BiO‐Logic instruments VSP potentiostat under dark conditions at room temperature with the same frequency in 200 kHz under applied bias (0.0−1.0 V). The *V*_bi_ and doping density (*N*) were calculated using the following equation,

(1) $$\frac{1}{C^{2}}=\frac{2(V_{\mathrm{bi}}-V)}{A^{2}q\varepsilon \varepsilon_{0}N}$$

where *C* is the capacitance, *A* is the active area (0.0512 cm^2^), *V* is the applied bias, *ε*_0_ is the vacuum permittivity, and the relative dielectric constant ε of PbS CQD is = 18.7.^[^ ^43^ ^]^ The *N*_t_ were calculated from the logarithm of the *I–V* curve obtained in the dark. SCLC techniques were performed by fabricating electron‐only devices (ITO/ZnO/PbS CQD/Au) to analyze the trap‐state density after ACA treatment. The *N*_t_ of the devices was estimated from the following equation

(2) $$V_{\mathrm{TFL}}=\;\frac{qN_{t}L^{2}}{2\varepsilon \varepsilon_{0}}$$

where *V*_TFL_ is the trap‐filled‐limit voltage (obtained from the SCLC curve), *q* is the elementary charge, and *L* is the film thickness. SEM was measured to get cross‐sectional image of device using a Hitachi S‐4800. The ns–μs transient absorption (TA) data were collected using a pump–probe TA spectroscopy, which consisted of a TA spectrometer and a regenerative amplified Nd:YAG laser (EL–YAG) with a pulse width of 6–8 ns. The pulse is capable of generating both visible pulses (532 nm) and UV pulses (355 nm) through a third harmonic generator. The TA spectra data were collected over a time range from 6 to 500 ns. The probe beam is derived from a 150 W Xenon lamp, which is reflected off the nanoparticle sample and then passed through a monochromator before reaching a PMT‐980 photodiode detector. To simultaneously capture data on two different time scales, the comprehensive L900 spectrometer software (V9.4.3) package is utilized, and the nanosecond‐microsecond signal is sampled using an oscilloscope (Tektronix MDO30232, Beaverton, OR, USA). Excitation fluences are measured using a pyroelectric energy sensor. EIS measurement was performed at 0.8 sun in the frequency range of 100 to 1 MHz, and the applied bias voltage was set as 0.3 V. The TPV and TPC were performed using a Nd:YAG laser (SLII‐10) excitation source tuned to a wavelength of 532 nm (10 Hz, with pulse duration <4 ns) as perturbation light and an Xe lamp (150 W, ABET tech) as a bias light source. The intensity of the laser was adjusted to keep ΔV smaller than 40 mV. The TPV and TPC data were fitted by the mono‐exponential method as a function of time for charge recombination and charge extraction.

(3) $$I(t)=A_{1}\exp\left[-\left(\frac{t}{\tau }\right)\right]$$

here, *I*(t) is the intensity as a function of time, *τ* is the charge lifetime, and *A*_1_ is the amplitude ratio.

## Supporting Information

Supporting Information is available from the Wiley Online Library or from the author.

## Conflict of Interest

The authors declare no conflict of interest.

## Author Contributions

**Eon Ji Lee**: conceptualization (lead); data curation (lead); formal analysis (lead); writing—original draft (lead). **Gayoung Ham**: data curation (lead); formal analysis (lead); investigation (lead); writing—original draft (equal). **Sunhee Yun**: formal analysis (supporting). **Hyung Ryul You**: investigation (supporting). **Taeyeong Yong**: investigation (supporting). **Gayoung Seo**: investigation (supporting). **Wonjong Lee**: investigation (supporting). **Hyeon Soo Ma**: investigation (supporting). **Jin Young Park**: investigation (supporting). **Hae Jeong Kim**: investigation (supporting). **Soo‐Kwan Kim**: investigation (supporting). **Younghoon Kim**: supervision (supporting). **Joungchul Lim**: investigation (supporting). **Minjun Kim**: investigation (supporting). **Hyojung Cha**: supervision (equal). **Jongmin Choi**: investigation (lead); supervision (lead); writing—original draft (lead). **Eon Ji Lee** and **Gayoung Ham** contributed equally to this work.

## Supporting information

Supplementary Material

## Data Availability

The data that support the findings of this study are available from the corresponding author upon reasonable request.
