# Supplementary material for: Enhancing Quantum Dot Photovoltaic Efficiency Through Defect Passivation and Triplet Energy Transfer with 9‐Anthracenecarboxylic Acid
Source: Small Sci. 2025 Aug 27;5(11):2500306. doi: 10.1002/smsc.202500306 (PMC12622402; doi:10.1002/smsc.202500306)
Supplement: Supplementary file 1 — Supplementary Material [file SMSC-5-2500306-s001.pdf]

Supporting Information

Enhancing Quantum Dot Photovoltaic Efficiency through Defect Passivation and Triplet Energy Transfer with 9-Anthracene Carboxylic Acid

Eon Ji Lee†, Gayoung Ham†, Sunhee Yun, Hyung Ryul You, Taeyeong Yong, Gayoung Seo, Wonjong Lee, Hyeon Soo Ma, Jin Young Park, Hae Jeong Kim, Soo-Kwan Kim, Younghoon Kim, Jongchul Lim, Minjun Kim, Hyojung Cha*, and Jongmin Choi**

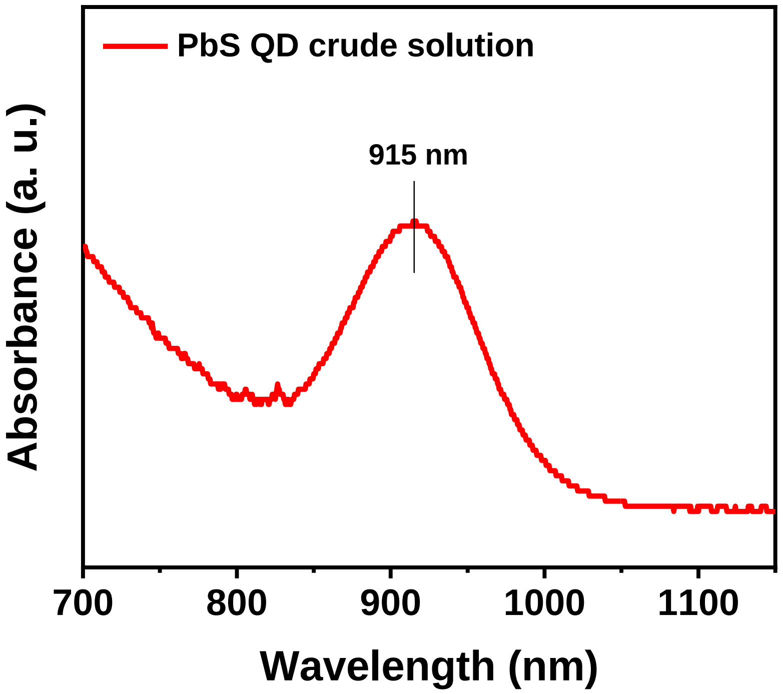

Figure S1. Absorbance spectrum of the oleate-capped PbS CQD solution in hexane.

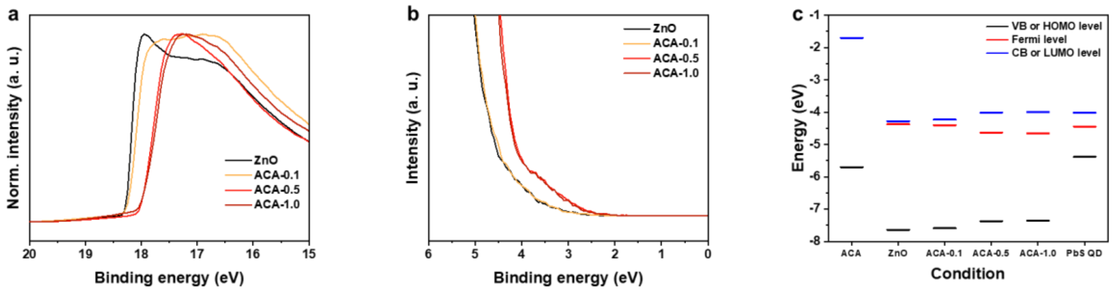

Figure S2. UPS spectra of ZnO, ACA-0.1, ACA-0.5, ACA-1.0, ACA, and PbS CQD films near the (a) secondary electron cut-off and (b) valence band region. (c) Overall energy level diagrams derived from UPS spectra and optical absorption bandgap. The HOMO and LUMO levels of ACA, as well as the energy levels of PbS CQD, were referenced from values reported in previous literature.[1,2]

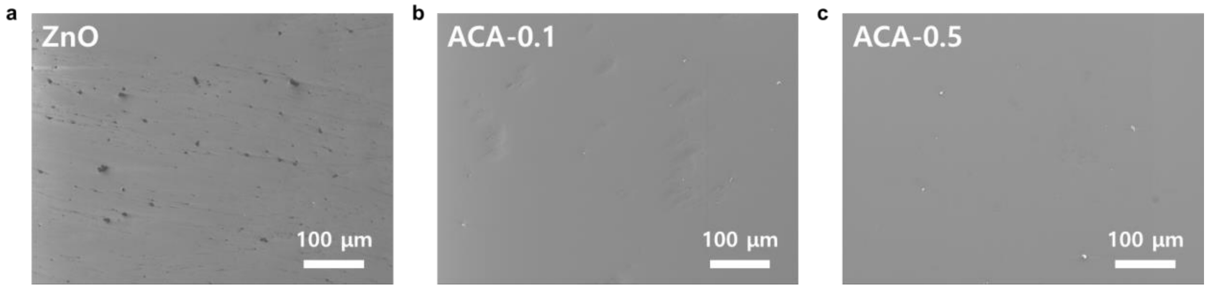

Figure S3. Top-view SEM images of films prepared under ZnO, ACA-0.1, and ACA-0.5 conditions.

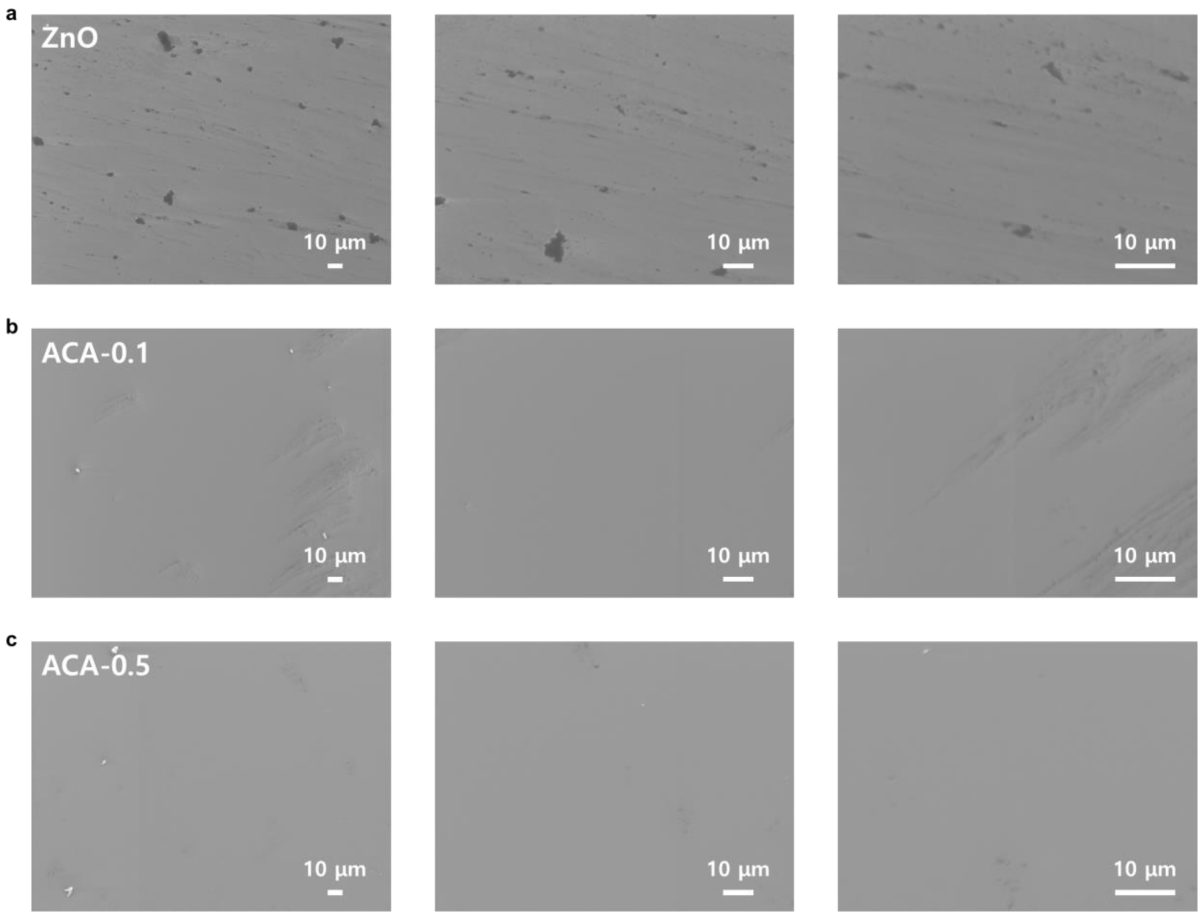

Figure S4. Top-view SEM images of films prepared under ZnO, ACA-0.1, and ACA-0.5 conditions, along with higher-magnification images.

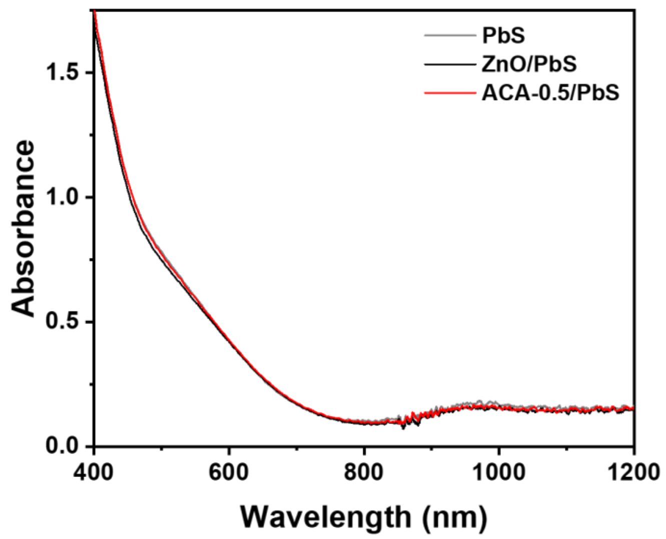

Figure S5. Absorption spectra of ETL/active layer bilayer films prepared under different processing conditions.

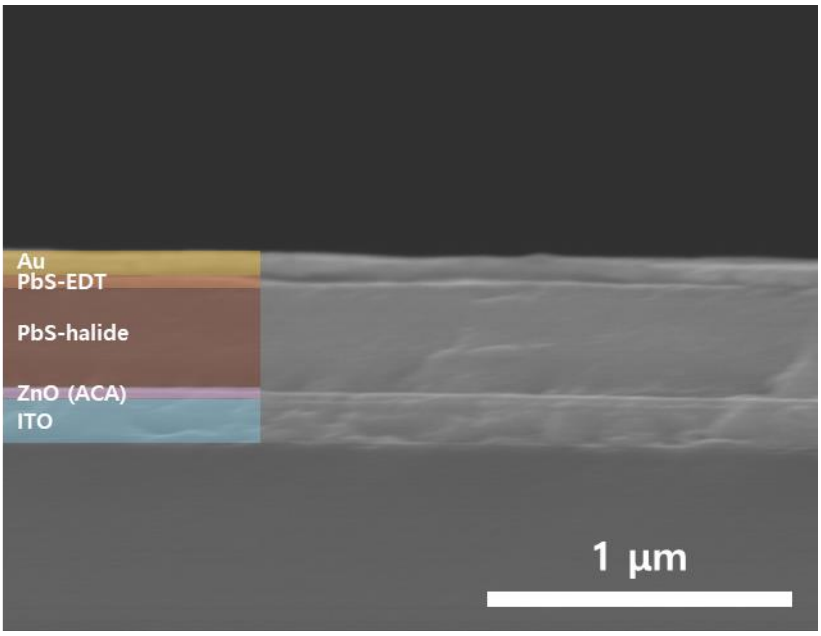

Figure S6. A Cross-sectional SEM image of the PbS CQD PVs. The thickness of the active layer was approximately ~325 nm.

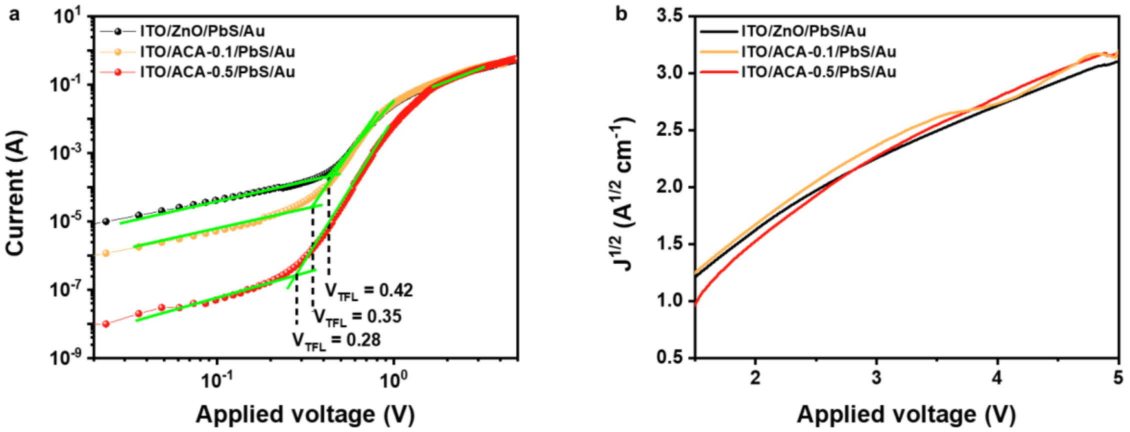

Figure S7. (a) SCLC spectra of pristine ZnO, ACA-0.1, and ACA-0.5-treated PbS CQD devices. (b) $J^{1/2}$-V curves from the (a) spectra.

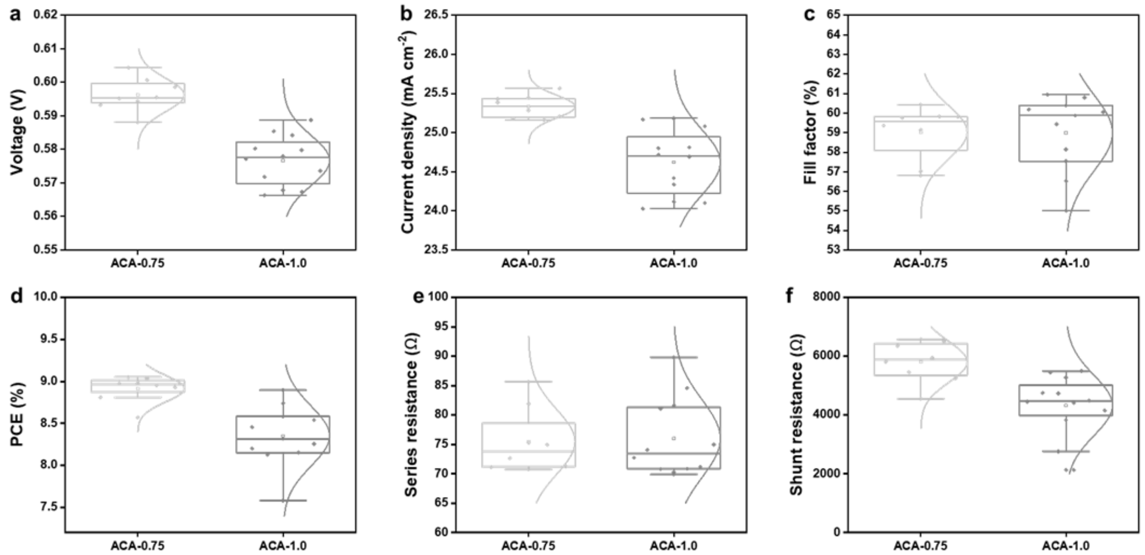

Figure S8. Full device histograms for 6~10 devices with ACA concentrations (0.75 and 1.0 mg mL^{-1}). (a) V_{oc}, (b) J_{sc}, (c) FF, (d) PCE, (e) series resistance, and (f) shunt resistance, respectively.

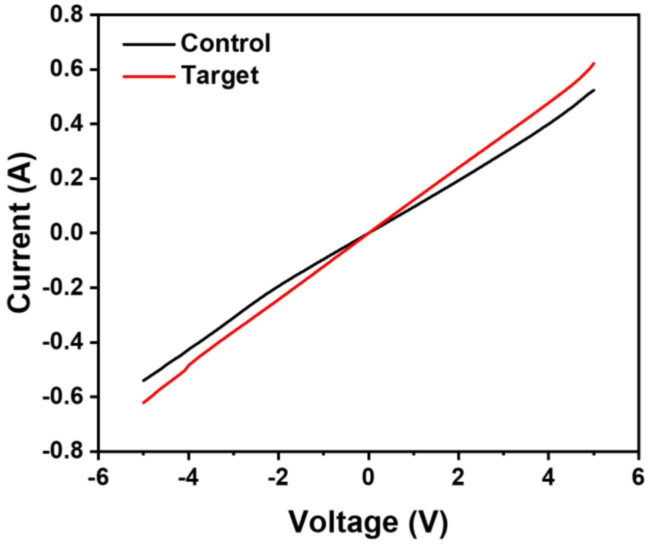

Figure S9. Conductivity spectra of ETL-only devices based on control (ZnO) and target (ACA-0.5).

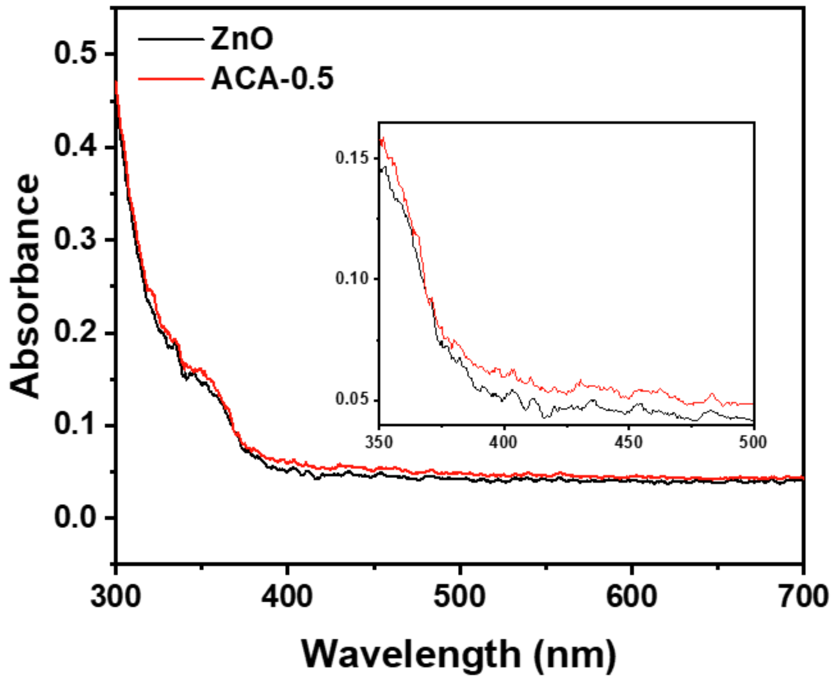

Figure S10. The absorbance spectra of ZnO and ACA-0.5 film, respectively. The inset shows a magnified spectrum view in the wavelength range of 350~500 nm.

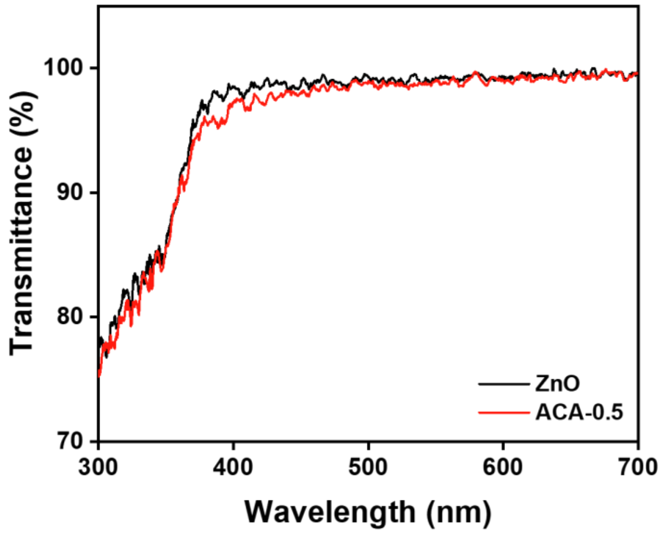

Figure S11. Transmittance spectra of the films coated with ZnO or ACA-0.5 on the glasses.

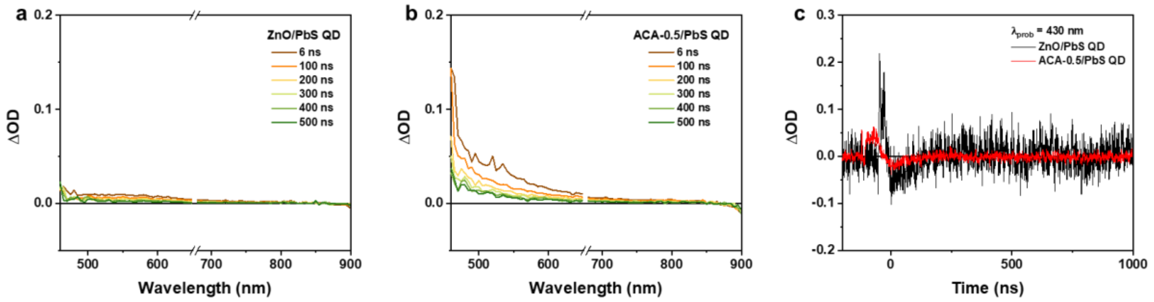

Figure S12. TA spectra of (a) ZnO/PbS CQD film, (b) ACA-0.5/PbS CQD film for time decays up to 500 ns, and (c) TA kinetics monitored with 430 nm probe wavelength.

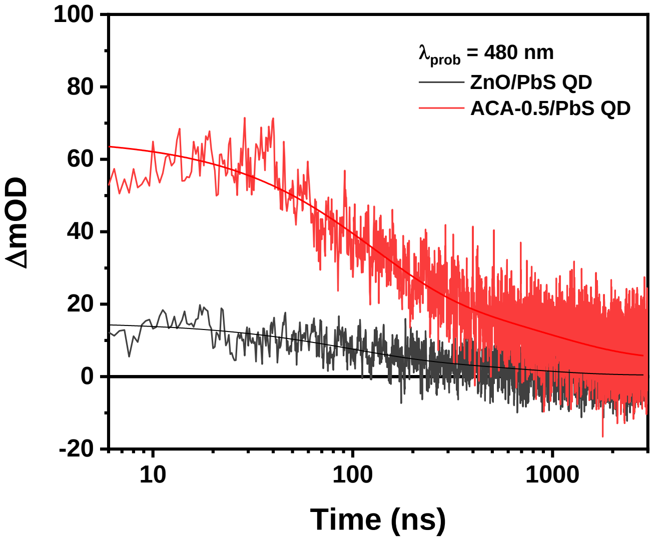

Figure S13. TA kinetics probed at 480 nm, lifetimes were calculated from the bi-exponential line.

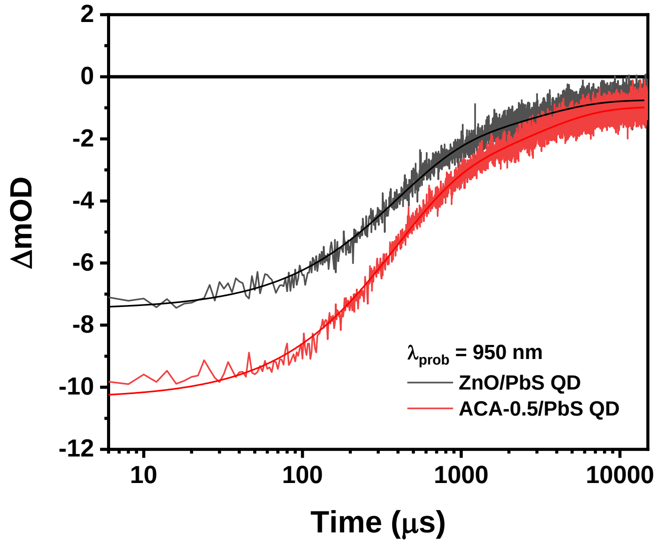

Figure S14. TA kinetics probed at 950 nm, lifetimes were calculated from the bi-exponential line.

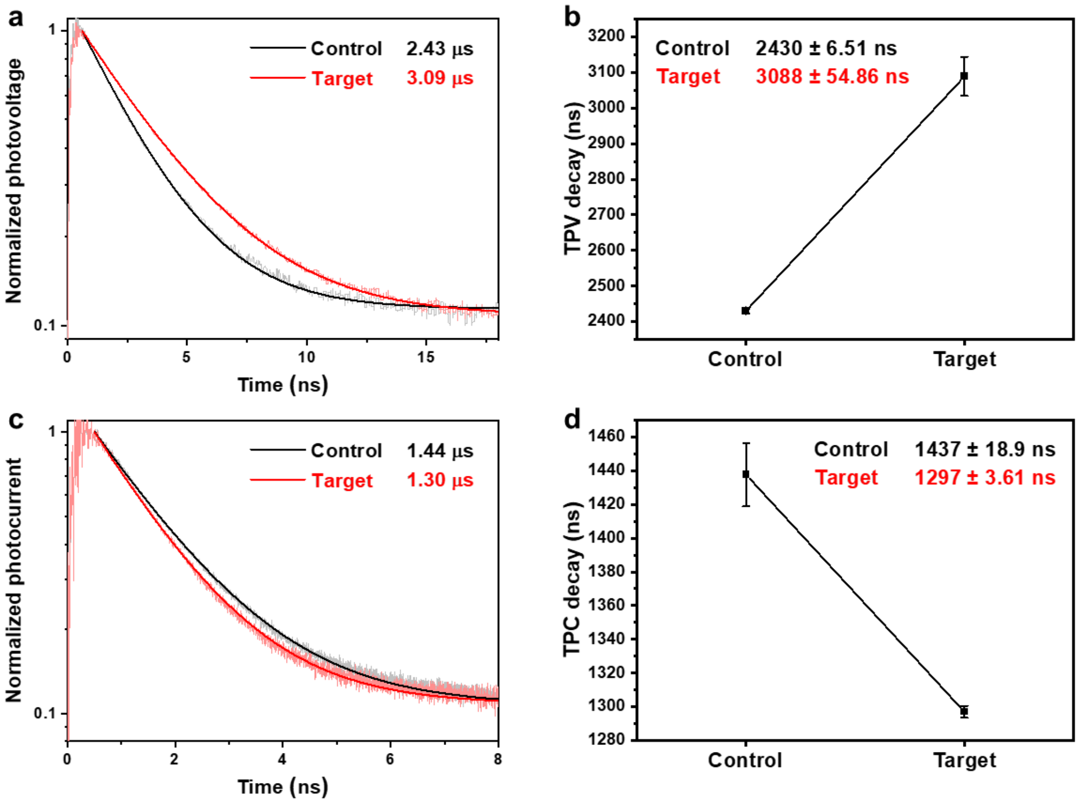

Figure S15. Charge carrier dynamics of the control and target devices. a) Normalized TPV, b) TPV decay, c) normalized TPC, and d) TPC decay spectra.

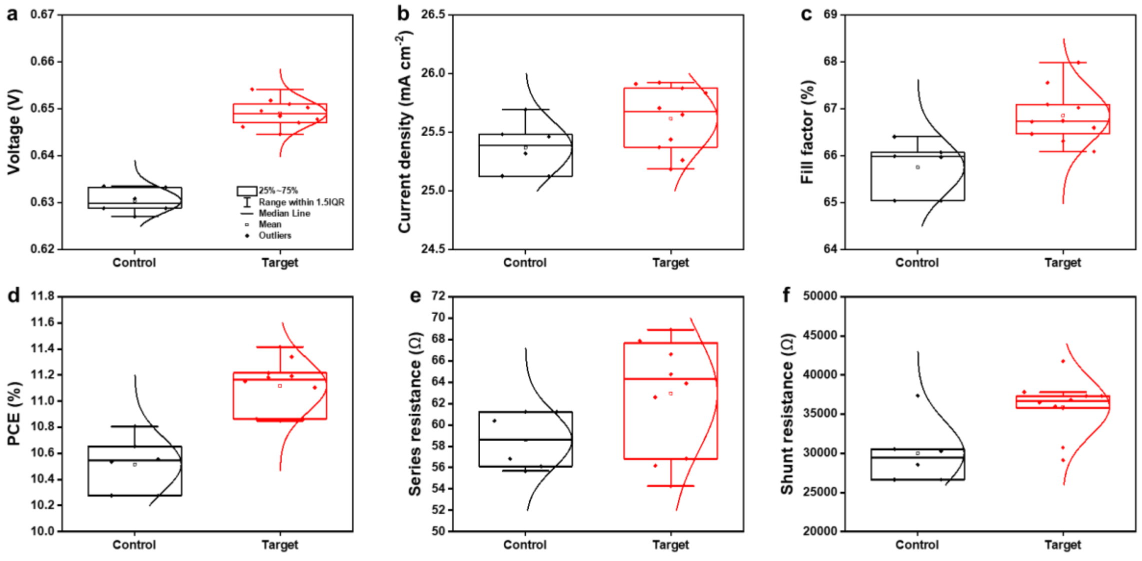

Figure S16. Device histograms for 6~10 devices with control and target devices. (a) V_{oc}, (b) J_{sc}, (c) *FF*, (d) *PCE*, (e) series resistance, and (f) shunt resistance, respectively.

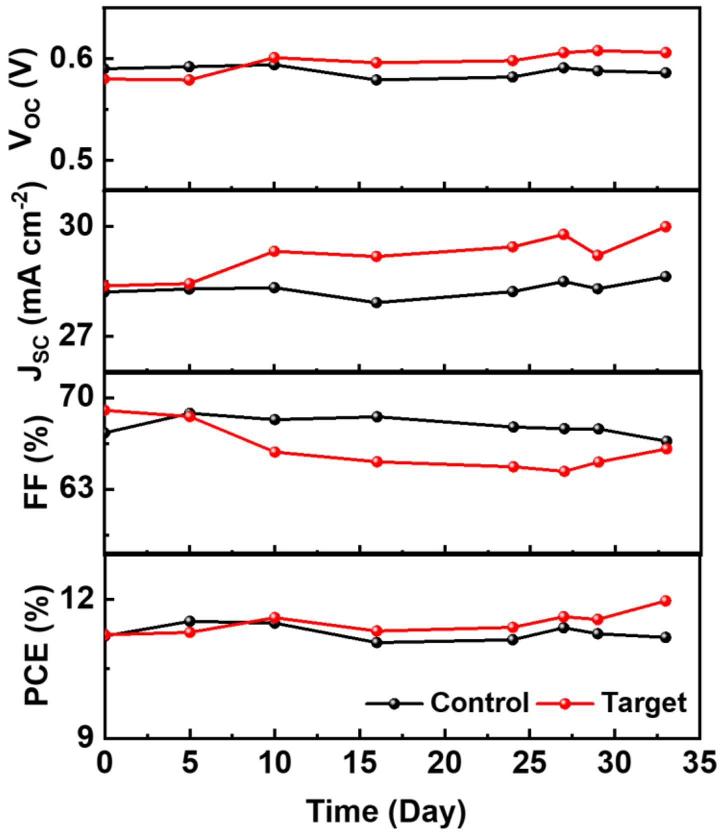

Figure S17. Stability graph of unencapsulated devices under ambient conditions for 33 days.

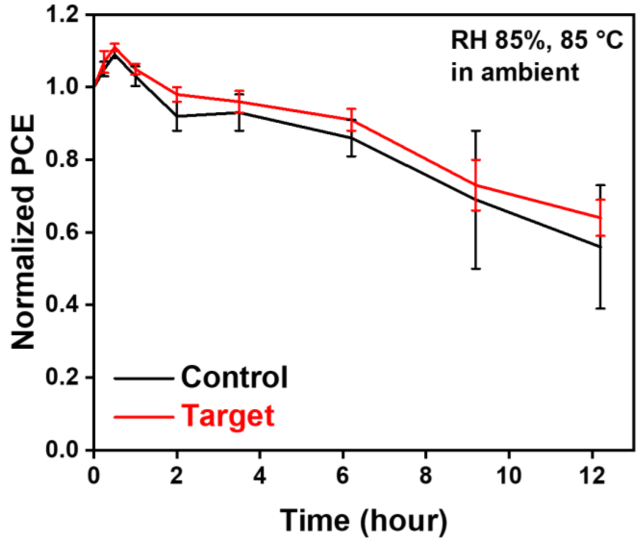

Figure S18. Stability test of PbS CQD PVs under thermal stress at 85 ℃ and 85% relative humidity (RH) in ambient air.

	Valence band or HOMO	Fermi level	Conduction band or LUMO
ACA[1]	-5.7		-1.7
ZnO	-7.64	-4.37	-4.28
ACA-0.1	-7.59	-4.4	-4.23
ACA-0.5	-7.37	-4.63	-4.01
ACA-1.0	-7.36	-4.65	-4.0
PbS CQD[2]	-5.38	-4.44	-4.01

Table S1. UPS energy level table of ACA, ZnO, ACA-0.1, ACA-0.5, ACA-1.0, and PbS CQD films from Figure S2.

	V_{TFL} (V)	Trap density (# cm^{-3})	Electron mobility (cm^2 V^{-1} s^{-1})
ZnO	0.42	7.85×10^{15}	1.84×10^{-2}
ACA-0.1	0.35	6.55×10^{15}	1.87×10^{-2}
ACA-0.5	0.28	5.24×10^{15}	2.42×10^{-2}

Table S2. Fitted parameters from the SCLC spectra (Figure S7) of pristine ZnO and ACA-treated devices.

	A1	τ_1 (ns)	A2	τ_2 (ns)	τ_{avg} (ns)
ZnO	0.0098	86.74	0.0047	664.9	274.1
ACA-0.5	0.0420	115.5	0.0188	952.5	374.3

Table S3. Lifetime TA kinetics parameters at 480 nm from the bi-exponential equation.

Equation : $y = y0 + A1 * exp\left(-\frac{x-x0}{t1}\right) + A2 * exp(-\frac{x-x0}{t2})$

	A1	τ_1 (ns)	A2	τ_2 (ns)	τ_{avg} (ns)
ZnO	-0.0052	366.6	-0.0017	2618	825.5
ACA-0.5	-0.0068	356.2	-0.0025	2734	995.3

Table S4. Lifetime TA kinetics parameters at 950 nm from the bi-exponential equation.

Equation : $y = y0 + A1 * exp\left(-\frac{x-x0}{t1}\right) + A2 * exp(-\frac{x-x0}{t2})$

	Series resistance (Ω)	Charge transfer resistance (Ω)	Recombination resistance (Ω)
**Control**	**21.40**	**82.59**	**147.11**
**Target**	**18.61**	**57.70**	**197.50**

Table S5. Fitted resistance parameters from the Nyquist plots shown in Figure 5b.

Reference list

[1] Ronchi et al., *Adv. Mater.*, **2020**, 32, 2002953

[2] Lee et al., *Small*, **2024**, 20, 2400380
